# Altered Gut Microbiota Composition in Subjects Infected With *Clonorchis sinensis*

**DOI:** 10.3389/fmicb.2018.02292

**Published:** 2018-09-28

**Authors:** Meng Xu, Zhihua Jiang, Wen Huang, Jianhai Yin, Shen Ou, Yanyan Jiang, Liyu Meng, Shengkui Cao, Aiping Yu, Jianping Cao, Yujuan Shen

**Affiliations:** ^1^National Institute of Parasitic Diseases, Chinese Center for Disease Control and Prevention, Shanghai, China; ^2^Chinese Center for Tropical Diseases Research, Shanghai, China; ^3^World Health Organization Collaborating Centre for Tropical Diseases, Shanghai, China; ^4^National Center for International Research on Tropical Diseases, Ministry of Science and Technology, Shanghai, China; ^5^Key Laboratory of Parasite and Vector Biology, Ministry of Health, Shanghai, China; ^6^Guangxi Zhuang Autonomous Region Center for Disease Control and Prevention, Nanning, China; ^7^Tengxian Center for Disease Control and Prevention, Tengxian, China

**Keywords:** fecal microbiota, microbiome, *Clonorchis sinensis*, 16S rRNA gene sequencing, dysbiosis

## Abstract

Clonorchiasis is an infectious disease caused by helminths of *Clonorchis sinensis* (*C. sinensis*). The adult parasite mainly inhabits the bile duct and gall bladder, and results in various complications to the hepatobiliary system. The amount of bile secreted into the intestine is reduced in cases of *C. sinensis* infection, which may alter the pH of the gut and decrease the amount of surfactant protein D released from the gallbladder. However, the impact of parasitic infection on the human gut microbiome remains unclear. To this end, we examined the gut microbiota composition in 47 modified Kato–Katz thick smear-positive (egg-positive) volunteers and 42 healthy controls from five rural communities. Subjects were grouped into four sub-populations based on age and infection status. High-throughput 16S rRNA gene sequencing revealed significant changes in alpha diversity between EP1 and EN1. The beta diversity showed alterations between *C. sinensis*-infected subjects and healthy controls. In *C. sinensis* infected patients, we found the significant reduction of certain taxa, such as *Bacteroides* and anti-inflammatory *Bifidobacterium* (*P* < 0.05). *Bacteroides*, a predominant gut bacteria in healthy populations, was negatively correlated with the number of *C. sinensis* eggs per gram (EPG, *r* = −0.37, *P adjust* < 0.01 in 20–60 years old group; *r* = −0.64, *P adjust* = 0.04 in the 60+ years old group). What’s more, the reduction in the abundance of *Bifidobacterium*, a common probiotic, was decreased particularly in the 60 + years old group (*r* = −0.50, *P* = 0.04). The abundance of *Dorea*, a potentially pro-inflammatory microbe, was higher in infected subjects than in healthy individuals (*P* < 0.05). *Variovorax* was a unique bacteria that was only detected in infected subjects. These results clearly demonstrate the significant influence of *C. sinensis* infection on the human gut microbiota and provided new insights into the control, prevention, diagnosis, and clinical study of clonorchiasis through the human gut microbiota.

## Introduction

*Clonorchis sinensis*, the Chinese liver fluke, is a key foodborne zoonotic pathogen that is frequently infects human via contaminated raw or undercooked fish (Choi et al., 2006; Qian et al., 2016). Adult *C. sinensis* is mainly found in the bile duct and gall bladder and effects the digestive system (Qian et al., 2016). Symptoms include diarrhea, nausea, indigestion and abdominal pain, cholelithiasis, cholangitis, and cholecystitis (Qian et al., 2016; Huang et al., 2017). Chronic *C. sinensis* infection has been shown to cause cholangiocarcinoma (CCA) (Brindley et al., 2015; Prueksapanich et al., 2018). In 2009, *C. sinensis* was classified as a group 1 biological carcinogen by the International Agency for Research on Cancer (Sripa et al., 2012). Currently, about 200 million individuals are at risk of infection with *C. sinensis* (Tang et al., 2016), predominantly in the eastern regions of Asia (i.e., China, Korea, and northern Vietnam), where individuals habitually eat raw or undercooked freshwater fish (Choi et al., 2006). More than 4,700 CCA patients have been found to be infected with *C. sinensis* (Qian et al., 2016; Qian and Zhou, 2017). Clonorchiasis is a major health problem in endemic areas, which has led to the loss of about 275,370 disability-adjusted life years (DALYs) (Qian et al., 2016). However, due to its prolonged inhabitation of the bile duct, the misdiagnosis of clonorchiasis (Lun et al., 2005), and the dietary habits of humans, the current control and prevention strategies for *C. sinensis* have been unable to completely eliminate the pathogen. Thus, to derive improved control, prevention, and treatment strategies, it is essential to further understand the infection mechanism of *C. sinensis* pathogens and its interactions with the host.

The human intestinal microbiota is initially established within 10 days of birth (Sommer and Bäckhed, 2013), and the composition, diversity, and function of the microbiota reaches maturity after 2 or 3 years (Yatsunenko et al., 2012). The gut microbiome can encode approximately 100 times more genes than the human genome (O’Hara and Shanahan, 2006), and contains a large metabolic capacity (Gill et al., 2006; O’Hara and Shanahan, 2006), which is essential to maintaining homeostasis in the gut. Furthermore, the gut microbiota plays an important role in maintaining the integrity of the mucosal barrier and modulating immune defense (Gensollen et al., 2016); these can confer protection against pathogen colonization (Thursby and Juge, 2017; Vogt and Finlay, 2017). Dysbiosis of the gut microbiota can increase susceptibility to infections and inflammatory diseases (Sirisinha, 2016; Thursby and Juge, 2017). Recently, host–parasite–microbiota relationships have emerged as a new research topic in the microbiome. Studies have shown that parasitic infection may adversely alter the composition of the gut microbiota (Iebba et al., 2016; Nagel et al., 2016; Beghini et al., 2017; Jenkins et al., 2017; Wang S. et al., 2017; Aksoy, 2018; Rosa et al., 2018) and accelerate disease processes (Azambuja et al., 2005; Villarino et al., 2016; Cai et al., 2017; Wang Q. et al., 2017). However, dysbiosis of the gut microbiota in intermediate hosts may influence the growth of parasites and weaken their dissemination (Villarino et al., 2016; Aksoy, 2018).

*C. sinensis* resides in the bile duct and completely consume bile acids, suppressing the release of bile from the gallbladder into the intestines. This may inhibit fat digestion by the host, alter the pH of the gut, and modulate gut microbiota composition. Current information regarding the impact of *C. sinensis* infection on the gut microbiota is based on animal models. In *C. sinensis* infected mice, the relative abundances of *Lactobacilli* and *Bifidobacteria* decreased; meanwhile, the relative abundances of *Enterobacter* and *Enterococci* increased (Cai et al., 2017). Another study showed that *C. sinensis* infection led to translocation of bacteria from the intestine to the liver in mice (Wang S. et al., 2017). Yet another study showed that surfactant protein D, which is synthesized in the gallbladder and delivered to the intestine, can regulate the symbiosis of the gut microbiota to maintain homeostasis (Sarashina-kida et al., 2017). All of these changes suggest an alteration to the intestinal microbiota with *C. sinensis* infection. Diet is considered a main factor that on influences the composition of the gut microbiota (Thursby and Juge, 2017). Previous studies have indicated that nutritional interventions may enhance human health and the relative stability of the gut microbiota, which may prevent parasitic infection (Knutie et al., 2017) and be beneficial for controlling the spread of parasites and disease prevention (Glendinning et al., 2014). We found a correlation between alterations in gut microbiota composition and the number of *C. sinensis* eggs per gram (EPG, an estimation of the number of eggs per gram of stool). These findings provide a foundation for the development of novel approaches to control and prevent *C. sinensis* infection, as well as novel treatments for preventing clonorchiasis progression, such as probiotic use or fecal microbiota transplantation (FMT).

## Materials and Methods

### Ethics Statement

Ethical clearance for the collection and examination of human fecal samples was approved by the Ethics Committee of the National Institute of Parasitic Diseases, Chinese Center for Disease Control and Prevention, China (No. 201401) on July 14, 2014. All participants knew the objectives, procedures, and potential risks of the study, and gave informed consent.

### Subjects and Methods

A cross-sectional study was carried out in October 2016 in Teng County, Guangxi Zhuang Autonomous Region, China. A total of 1,000 fresh stool samples from volunteers were collected in the morning for triplicate modified Kato-Katz thick smear analysis (Hong et al., 2003), and counted for infection intensity (estimated using EPG). EPG was calculated as: EPG = N^∗^24 where N = the mean of triplicate *C. sinensis* eggs counts for each 41.67 mg stool sample, and 24 is a conversion factor (Chen et al., 2018).

A total of 89 individuals were enrolled in this study based on the results of the modified Kato-Katz thick smear method, including 42 healthy controls and 47 *C. sinensis* egg positive subjects (infectious status by EPG: mild infection and moderate infection) (Chen et al., 2018). The 89 fresh fecal samples were collected in a sterile tub, and placed on ice. All samples were immediately transported to the laboratory after collection. The samples were collected in duplicate, including one for microscopic examination and for sequencing, which was immediately stored at −80°C in a sterile, labeled Eppendorf tube for DNA extraction. No other parasites eggs (e.g., *horkworms*, *roundworm*, *whipworm*, *pinworm*, *Taenia*) were detected by microscopic examination in 47 *C. sinensis* egg positive subjects fecal samples. Detailed clinical parameters/demographics for the 89 participants are shown in **Supplementary Table S1**.

EPG is used to characterize *C. sinensis* infection (Vlčková et al., 2018). Based on EPG and age, the 89 subjects in this study were assigned to one of four groups:

(1) egg positive between 20 and 60 years old (EP1, EPG≥24, *n* = 38);
(2) egg negative between 20 and 60 years old (EN1, EPG = 0, *n* = 34);
(3) egg positive over 60 years old (EP2, EPG≥24, *n* = 9), and;
(4) egg negative over 60 years old (EN2, EPG = 0, *n* = 8).

All of the study participants were from the same geographical area, over 20 years old, and with no other parasitic infections; they had no received antibiotic treatments for at least 3 months prior to being enrolled. A total of 89 fresh fecal samples were stored in 2.0 ml Eppendorf tubes and frozen at −80°C for DNA extraction.

### DNA Isolation

Total bacterial genomic DNA was extracted from fecal samples using the QIAamp^®^DNA Stool Mini Kit (QIAGEN, Hilden, Germany) according to the manufacturer’s instructions. PCR amplification of the V3–V4 region of the 16S rRNA gene was performed using the 338F forward primer (5′-ACTCCTACGGGAGGCAGCA-3′) and the 806R reverse primer (5′-GGACTACHVGGGTWTCTAAT-3′). The PCR cycle was denaturation at 98°C for 2 min, followed by: 25 cycles of denaturation at 98°C for 15 s, annealing at 55°C for 30 s, and extension at 72°C for 30 s; then, there was a final extension at 72°C for 5 min. PCR products were sequencing on an Illlumina MiSeq with the MiSeq Reagent Kit v3 at Shanghai Personal Biotechnology Co. Ltd (Shanghai, China).

### Sequence Analysis

The analysis of sequence reads was conducted using Quantitative Insights Into Microbial Ecology (QIIME, v1.8.0) (Caporaso et al., 2010). Briefly, raw sequences were matched to barcodes and identified as valid sequences. The primers sequences and barcodes were removed and sequences underwent quality control. The elimination criterion were: length <150 bp, average Phred scores of <20, ambiguous bases, and mononucleotide repeats of >8 bp. After chimera detection, the remaining high-quality sequences were clustered into operational taxonomic units (OTU) at 97% sequence identity by UCLUST (Edgar, 2010). To ensure the reliability and accuracy of the analysis, OTUs with abundances below 0.001% of all samples sequences were removed (Bokulich et al., 2013). To minimize the difference of sequencing depth across samples, an averaged, rounded rarefied OTU table was generated by averaging 100 evenly resampled OTU subsets under 90% of the minimum sequencing depth; this OTU table was used for further analysis.

### Bioinformatics and Statistical Analysis

Sequencing data were primarily analyzed using the Quantitative Insights Into Microbial Ecology (QIIME, v 1.8.0) and R packages (v 3.2.0). OTU-level species accumulation curves were used to estimate the sequencing depth and species richness. Alpha diversity indices, including Chao 1 richness, the ACE metric (Abundance-based Coverage Estimator), the Simpson index, and the Shannon diversity index, were calculated using QIIME. Kruskal–Wallis tests were used to identify significant differences in alpha diversity indexes between groups. Taxa abundances at the phylum and genus levels were analyzed using the Metastats function in Mothur and visualized as violin plots. Linear discriminant analysis effect size (LEfSe) was performed to detect differentially abundant taxa between groups using the default parameters (Segata et al., 2011), and the threshold logarithmic linear discriminant analysis (LDA) score was set to 2.0. Partial least squares discriminant analysis (PLS-DA), a supervised model to reveal microbiota variation between groups, was used to identify the key genera that were responsible for the differential microbiota structure using the “plsda” function in the R package “mix Omics” (Chen et al., 2011). In this study, the key genera with variable importance in projection (VIP) > 1.5 were considered to be important contributors to the model. Adonis/PERMANOVA tests were used to evaluate differences between groups. Spearman correlation analyses were used to determine correlations between gut microbiota composition and the number of EPG.

### Data Access

All raw sequences were deposited in the NCBI Sequence Read Archive (accession number SRP158183).

## Results

### Changes in Overall Microbial Community Diversity and Structure in *C. sinensis* Infected Subjects

A total of 4,386,958 valid sequences were generated from 89 human fresh fecal samples, and after quality filtering and trimming, the remaining 3,190,086 high-quality sequences were acquired, with an average of 35,844 sequences each samples (**Supplementary Table S2**). All high-quality sequences were clustered into OTUs at 97% sequence identity. After discarded the low-credibility OTUs (containing less than 0.001% of total sequences across all samples), a modified OTU table was obtained consisting of 3,023 OTUs with an average of 848 OTUs per sample (ranging from 372 to 1,340; **Supplementary Tables S2**, **S3**).

The species accumulation curves showed a plateau and a saturation phase (**Supplementary Figures S1A,B**); this indicates that the sample size was sufficient to capture the overall microbiota structure.

The Simpson index was significantly different between EN1 and EP1 groups (Kruskal–Wallis test, *P* = 0.038). There were no significant differences in estimated OTU richness, Chao1, the ACE metric, the Shannon diversity index, Good’s coverage, or the Simpson index (60+ age groups) between groups (*P* > 0.05, **Table 1**).

**Table 1:** Alpha diversity of the gut microbiota in the different groups.

Group	Chao1		ACE		Shannon		Simpson		Coverage	
	Mean	*SE*	Mean	*SE*	Mean	*SE*	Mean	*SE*	Mean	*SE*
EN1	523.3	142.87	546.81	151.03	5.16	0.72	0.91^∗^	0.06	0.99	0.00
EP1	550.12	161.98	562.72	161.20	5.46	0.72	0.93	0.04	0.99	0.00
EN2	438.87	221.99	461.29	236.24	4.64	1.60	0.85	0.16	0.99	0.00
EP2	570.11	126.49	594.04	139.45	4.88	0.93	0.86	0.12	0.99	0.00

*SE, Standard Error. ^∗^Indicates significant different (P < 0.05). Simpson index between EN1 and EP1 was statistically significant different (P = 0.038).*

**FIGURE 1:**
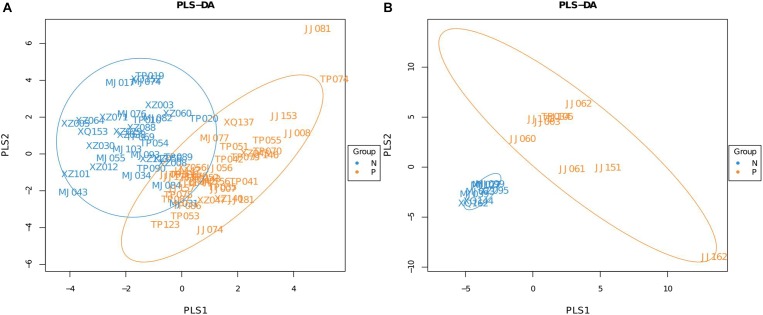
Partial Least Squares Discriminant Analysis (PLS-DA) of *C. sinensis* infected subjects and healthy controls. **(A)** Is the 20–60 years old group, and **(B)** is the over 60 years old group. Each point represents a sample and points with the same color belong to the same group. The blue points represent egg negative subjects and the yellow points represent egg positive subjects.

A Partial Least Squares Discriminant Analysis (PLS-DA), which is based on a least squares regression model, showed that there were structural differences in the gut bacterial community structure between the groups (**Figures 1A,B**). These differences were assessed by calculating UniFrac distances, a phylogenetic based distance metric which, when weighted, accounts for the relative abundance of taxa (Karagiannis-Voules et al., 2015). An adonis analysis based on the unweighted UniFrac distances found that in both age groups the microbiota composition of subjects that were egg positive was significantly different from subjects that were egg negative (*P* = 0.037 for the 20–60 years old groups and *P* = 0.01 for the over 60 years old group). However, using the weighted UniFrac distances, only the 20–60 years old groups were significantly different (*P* = 0.002).

### Differences in Bacterial Abundance Between the EP1 and EN1, and the EP2 and EN2 Groups

**FIGURE 2:**
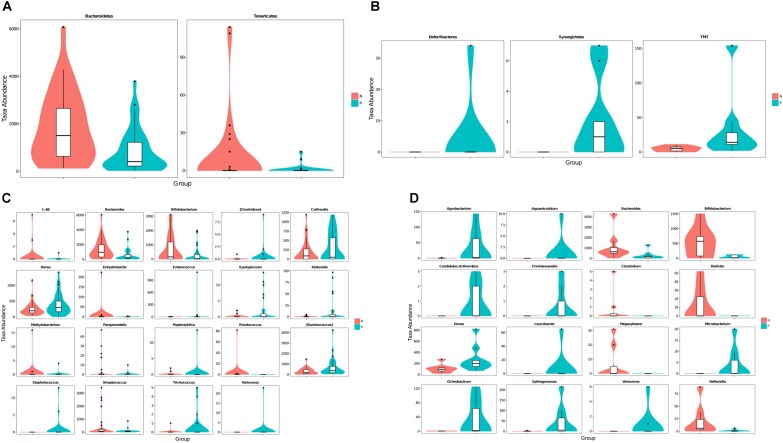
Violin plots of differentially abundant taxa between the *C. sinensis* infected subjects and healthy controls. (**A,C**) Are the 20–60 years old group (**A**: phylum level, **C**: genus level); (**B,D**) are the over 60 years old group (**B**: phylum level, **D**: genus level). *C. sinensis* infected subjects are shown in green and healthy controls are shown in red. The horizontal coordinates are the first 20 phyla or genera that are most significantly different and the vertical coordinates are the sequence quantities of the corresponding taxa for each group. The thickness of the violin plot reflects the density of the sample distribution and the box border represents the interquartile range (IQR).

Taxa abundances at the phylum and genus levels were compared using Metastats. The relative abundance of *Tenericutes* and *Bacteroidetes* were significantly decrease in EP1 group (**Figure 2A**). Meanwhile, *Deferribacteres* and *Synergistetes* were only detected in EP2 (**Figure 2B**).

A Metastats analysis of bacterial abundances at the genus level showed 19 differentially abundant genera in the 20–60 years old population (**Figure 2C**). Meanwhile, there were 16 differentially abundant genera in the 60+ years old population (**Figure 2D**). The relative abundances of *Bacteroides* and *Bifidobacterium* were lower in the *C. sinensis*-infected group than in the healthy controls; this corresponded to an increased relative abundance of *Dorea*. Interestingly, the genus *Variovorax* was only found in *C. sinensis* infected group. In the 20–60 years old population, *Staphylococcus* was only detected in EP1 group; meanwhile, *Rhodococcus* was not detected in this group. In the 60+ years old population, *Aquamicrobium*, *Leucobacter*, *Candidatus. Arthromitus*, *Christensenella*, and *Microbacterium* were only detected in the EP2 group; meanwhile, *Megasphaera, Clostridium*, and *Dialister* were only detected in the EN2 group. These differentially abundant taxa were further analyzed using LEfSe and PLS-DA analysis.

### Distinct Composition of the Gut Microbiota in *C. sinensis* Infected Subjects

**FIGURE 3:**
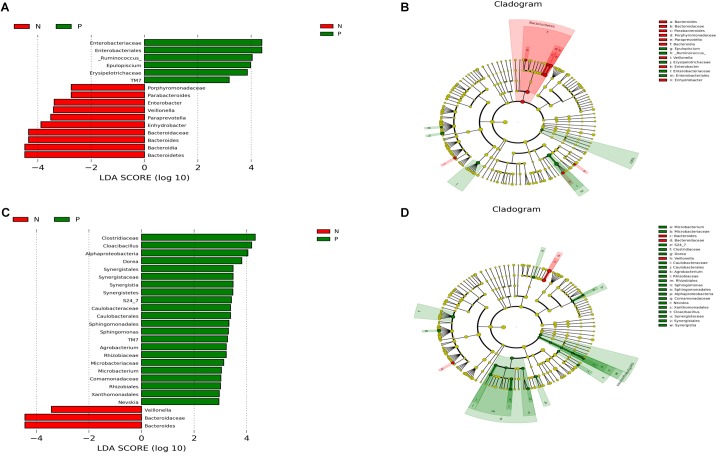
Comparison of microbiota variation at the genus level using the LEfSe online tool. **(A,C)** Are histogram of the LDA scores for differentially abundant taxa between groups (logarithmic LDA score threshold for discriminative features was set to 2.0). **(B,D)** Are cladograms showing taxonomic representations of significant differences between groups. **(A,B)** Are the 20–60 years old groups, and **(C,D)** are the 60+ years old groups. Red indicates that the taxa was more abundant in the healthy controls, and blue indicates that the taxa was more abundant in the **(C)**. sinensis infection group.

There were significant differences in the microbial community composition between the *C. sinensis*-infected group and the healthy controls (**Figure 3**). In the 20–60 years old group, *TM7* was significantly enriched in EP1 group; meanwhile, the abundance of *Bacteroidetes* was significant decreased in EP1 group. There were eight significantly different genera between groups. *[Ruminococcus]* and *Epulopiscium* were more abundant in EP1 group; meanwhile, *Paraprevotella*, *Bacteroides*, *Enhydrobacter*, *Parabacteroides*, *Enterobacter*, *Veillonella* were less abundant in the EP1 group. In the 60 + age group, *TM7* was more abundant in the EP2 group; this is consistent with the results from the 20 to 60 years old group. At the phylum level, *Synergistetes* was more abundant in the *C. sinensis* infected group. At the genus level, *Bacteroides* and *Veillonella* were less abundant in the EP2 group; meanwhile, *Nevskia*, *Sphingomonas*, *Agrobacterium*, *Cloacibacillus*, *Microbacterium*, *Dorea* were more abundant in the EP2 group.

In this study, we also used Partial Least Squares Discriminant Analysis (PLS-DA) model and used the VIP score to assess the contribution of each genus to the discrimination between the *C. sinensis* infected groups and healthy controls. A total of 15 genera with VIP > 1.5 were identified as key genera in the 20–60 years old group (**Table 2**); of these, 10 were differentially abundant between the EP1 and EN1 groups (**Figure 2C**). *Bacteroides* and *Bifidobacterium* had the highest VIP scores (VIP > 2.5). In the over 60 years old group, there were 13 key genera with VIP > 1.5 (**Table 2**); of these, eight were differentially abundant between the two groups (**Figure 2D**). In both age groups, *Bifidobacterium* had the highest VIP score (VIP = 2.51 in the 20–60 years old group; VIP = 2.23 in the 60+ years old group).

**Table 2:** The key differentially abundant taxa according to the PLS-DA model.

The age from 20 to 60		The age over 60	
Bacteria	VIP score	Bacteria	VIP score
*Bacteroides*	3.02	*Bifidobacterium*	2.23
*Bifidobacterium*	2.51	*Cloacibacillus*	1.82
*Streptococcus*	2.37	*Dorea*	1.71
*Paraprevotella*	2.32	*Dialister*	1.69
*Epulopiscium*	2.31	*Veillonella*	1.65
*Klebsiella*	2.29	*Ochrobactrum*	1.61
*[Ruminococcus]*	2.28	*Candidatus Arthromitus*	1.60
*Oscillospira*	2.08	*Bacteroides*	1.58
*Dorea*	2.07	*Streptococcus*	1.56
*Trichococcus*	1.90	*Agrobacterium*	1.55
*Acinetobacter*	1.75	*Pseudomonas*	1.55
*Odoribacter*	1.73	*[Eubacterium]*	1.51
*Collinsella*	1.57	*Granulicatella*	1.51
*Coprococcus*	1.56	–	–
*Slackia*	1.51	–	–

### Differences in Gut Microbiota Composition Are Correlated With EPG Levels

To further understand the variation of the fecal microbiota in *C. sinensis* infected subjects, we also evaluated the relation between EPG and gut microbiota composition (**Table 3**).

**Table 3:** Correlations between microbial taxa and EPG.

The age from 20 to 60				The age over 60			
Bacteria	*P*	*P. adjust*	*r*	Bacteria	*P*	*P*. *adjust*	*r*
*Bacteroides*	0.00	0	−0.37	*Bacteroides*	0.01	0.04	−0.64
*Parabacteroides*	0.01	0.03	−0.31	*Veillonella*	0.03	0.04	−0.53
*Oscillospira*	0.01	0.03	−0.29	*Bifidobacterium*	0.04	0.04	−0.50
*Veillonella*	0.02	0.04	−0.28	*Clostridium*	0.04	0.04	−0.35
*Paraprevotella*	0.03	0.04	−0.26	*Atopobium*	0.04	0.04	0.50
*Haemophilus*	0.04	0.04	−0.24	*Actinomyces*	0.02	0.04	0.55
*Enterobacter*	0.02	0.02	0.21	*Granulicatella*	0.02	0.04	0.57
*Trichococcus*	0.04	0.04	0.24	–	–	–	–
*Collinsella*	0.03	0.039	0.25	–	–	–	–

*P < 0.05 indicates that the genus has a correlation with EPG. P > 0.05 indicates that there is no correlation with EPG. r > 0 indicates that the genus has a positive correlation with EPG. r < 0 indicates that the genus has a negative correlation with EPG.*

In the 20–60 years old group, a total of nine genera were correlated with EPG. *Bacteroides, Parabacteroides, Oscillospira, Veillonella, Paraprevotella*, and *Haemophilus* had weak negative correlations with EPG (**Table 3**); meanwhile, *Enterobacter*, *Trichococcus*, and *Collinsella* were positively correlated with EPG (**Table 3**). In the over 60 years old group, there were seven genera that had a correlation with EPG. *Bacteroides, Veillonella, Clostridium*, and *Bifidobacterium* had a slight negative correlation with EPG (**Table 3**); meanwhile, *Atopobium, Actinomyces*, and *Granulicatella* had a weak positive correlation with EPG (**Table 3**).

## Discussion

In this study, we used high-throughput 16S rRNA gene sequencing to examine alterations in the gut microbiota in *C. sinensis* infection populations and explore the relationship between gut microbiota and EPG.

By comparing the alpha diversity index using Kruskal-Wallis, Simpson index showed significant higher in the EP1 group than in the EN1, which indicating that *C. sinensis* may increase the species diversity of human gut bacterial. This result was consistent with previous studies of the gut microbiota in soil-transmitted helminths (including hookworms and/or roundworms and/or whipworms) infected subjects (Lee et al., 2014; Rosa et al., 2018). However, in other studies, there were no significant increase in community richness in subjects infected with soil-transmitted helminths (Duarte et al., 2016; Jenkins et al., 2017) or intestinal protozoa (Barash et al., 2017). All of this difference results could be due to differences in infection status, geographical region, the specific parasite infection, the platform used, or the sequencing depth.

Adult *C. sinensis* inhabit the bile duct and can cause the inflammation of the bile duct walls and induce the expression of the pro-inflammatory cytokines (Qian et al., 2016). This may cause lead to alterations in the composition of the gut microbiota. We observed distinct microbial communities within the different groups. *Dorea* is a potentially pro-inflammatory taxa (Diling et al., 2017), and was significantly increased in *C. sinensis* infected subjects. This increase was also observed in other helminth infected individuals (Duarte et al., 2016). At the genus level, the abundance of *Bacteroides* and *Bifidobacterium* were significant decreased in the *C. sinensis* infected groups based on a Metastats analysis. In previous studies, the lower abundance of *Bacteroides* was also observed in parasites-positive samples (Duarte et al., 2016; O’Brien Andersen et al., 2016). *Bacteroides* are ubiquitous in the human gut and perform a number of metabolic functions, including the conversion of plant sugars to fermentation products, the remove of side chains from bile acids, and the return of bile acids to hepatic circulation. It is possible that *C. sinensis*, which resides in the bile duct, could alter the secretion of bile acids into the intestinal tract and alter the intestinal microbial community structure. We observed that this taxa had a weak negative correlation with EPG, which suggests that *C. sinensis* infection may cause a decrease in the relative abundance of *Bacteroides.* The LEfSe analysis results also indicated that *Bacteroides* is a key taxa that drives the differences in gut microbiota composition in *C. sinensis*-infected individuals. *Bifidobacterium*, an important probiotic bacteria, is considered a potentially anti-inflammatory taxa (Mokrozub et al., 2015) that can rebalance the gut microbiota to alleviate gastrointestinal symptoms (Vitetta et al., 2014). This taxa was significantly decreased in *C. sinensis*-infected populations. A previous study observed a decrease in the relative abundance of *Bifidobacteria* and *Lactobacilli* in rats infected with *C. sinensis*, which corresponded to an increase in the relative abundance of *Enterobacter* and *Enterococci* (Wang S. et al., 2017). In *Giardia*-infection rats, a higher abundance of *Lactobacilli* was detected (Pavanelli et al., 2018). We did not observe any significant alterations in the relative abundance of *Lactobacilli*. In other parasites-infected subjects, there were also no reported alterations in the relative abundance of *Lactobacilli* (Iebba et al., 2016; Jenkins et al., 2017). These inconsistent results may be based on the differences in parasites species, the differences in host organism, and/or the severity of the infection. The relative abundance of *Enterococcus* was higher in the EP1 group. *Enterococcus* is an important bacterial pathogen. Increased abundances of *Enterococcus*, along with increased antimicrobial resistance, have been observed with several pathogenic infections (Jia et al., 2014). In *C. sinensis*-infected individuals, the higher relative abundance of *Enterococcus* might not be conducive to recovery from clonorchiasis. *Variovorax*, an important environmental taxa (Thomas et al., 2017; Woo et al., 2017), has been reported to produce nitramine (Mahan et al., 2017), which is harmful to humans and animals. This taxa was only detected in the *C. sinensis* infected groups, however, it was not correlated with EPG. Eating raw or uncooked fresh fish is one of the main causes of *C. sinensis* infection. However, it is unclear whether the higher abundance of *Variovorax* in *C. sinensis*-infected individuals is related to eating habits; this should be investigated in future studies. *Staphylococcus*, an opportunistic pathogen, can cause a variety of diseases in humans and animals. Some subspecies of *Staphylococcus* have highly virulent pathogen (Plata et al., 2009) and multiple antibiotic-resistance (Gardete and Tomasz, 2014), which has been a therapeutical problem in clinical. In this study, This taxa was only detected in *C. sinensis* infected individuals in the 20–60 years old group, which may be detrimental to the treatment of the egg-positives. *Clostridium*, including common free-living bacteria (e.g., *Clostridium novyi*) and several significant human pathogens (e.g., *Clostridium botulinum*, *Clostridium difficile*), is a predominant genera in the human gut. In the over 60 years old groups, *Clostridium* was only found in health individuals and was negatively correlated with EPG. However, it is unclear whether *C. sinensis* infection influences specific species of *Clostridium* due to the sequencing depth.

In this study, *C. sinensis* infection did significantly alter the structure of the gut microbiota. This indicates that *C. sinensis* infection can cause dysbiosis. A previous study found that changes in gut microbiota composition can promote the formation of cholesterol gallstones in mice (Wang Q. et al., 2017). Cholelithiasis is one of the complications of *C. sinensis* infection (Xia et al., 2015). Weather Cholelithiasis in *C. sinensis* egg-positive individuals was relate to dysbiosis of gut microbiota was unclear. So, more extensive research is required to advance these ideas toward practice; our study provides a basis for further work in this area.

## Conclusion

This study provides a preliminary view of the effects of *C. sinensis* infection on the human gut microbiota in China. The structure of the gut microbiota was altered in *C. sinensis*-infected individuals, and some of these changes were associated with EPG. At this point, we cannot determine if these are causal relationships; this requires further studies. These results could be used to inform the diagnosis, prevention, and treatment of Clonorchiasis, although further studies are required before clinical implementation.

## Author Contributions

YS and JC conceived and designed this study. MX, ZJ, WH, JY, SO, YJ, LM, SC, and AY performed the experiments. MX, ZJ, and YJ analyzed the data. YS and JC contributed reagents and materials. MX, YS, and JC wrote the manuscript and prepared the tables and figures. All authors edited the manuscript.

## Conflict of Interest Statement

The authors declare that the research was conducted in the absence of any commercial or financial relationships that could be construed as a potential conflict of interest.

## Supplementary Material

FIGURE S1 Species accumulation curves. **(A)** Represents the 20–60 years old group and **(B)** represents the over 60 years old group. The curves reach a plateau and a saturation phase, which verifies that the sample size was sufficient to capture details of the microbial community.

TABLE S1 Clinical/demographic parameters of the 160 participants.

TABLE S2 Sequences and modified OTUs of 89 fecal samples.

TABLE S3 Modified OTU table at 97% identity.

## Abbreviations

*C. sinensis*: *Clonorchis sinensis*
DNA: deoxyribonucleic acid
EPG: eggs per gram
OTU: operational taxonomic units
PCoA: principal coordinates
QIIME: Quantitative Insights Into Microbial Ecology
rRNA: ribosomal ribonucleic acid analysis
VIP: the variable importance in projection.
