# Supplementary figures and images for: Altered Gut Microbiota Composition in Subjects Infected With Clonorchis sinensis
Source: Front Microbiol. 2018 Sep 28;9:2292. doi: 10.3389/fmicb.2018.02292 (PMC6172334; doi:10.3389/fmicb.2018.02292)

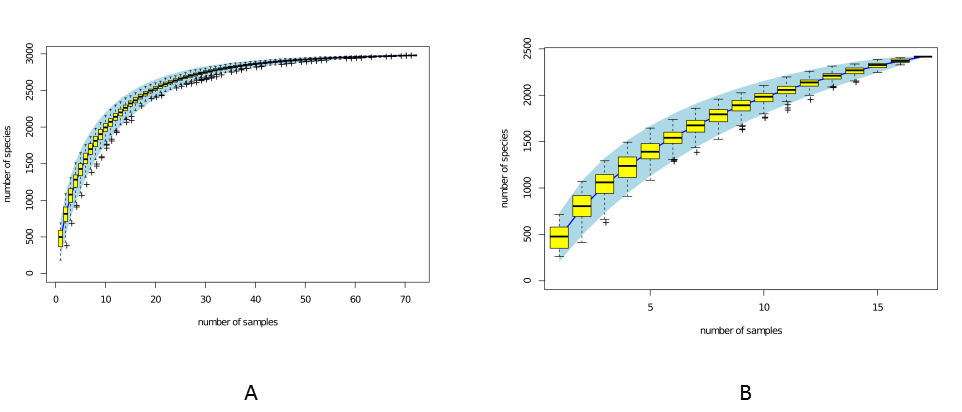

Supplement: FIGURE S1 — Species accumulation curves. (A) Represents the 20–60 years old group and (B) represents the over 60 years old group. The curves reach a plateau and a saturation phase, which verifies that the sample size was sufficient to capture details of the microbial community. [file Image_1.PNG]
